# Comparative studies of hair shaft components between healthy and diseased donors

**DOI:** 10.1371/journal.pone.0301092

**Published:** 2024-05-08

**Authors:** Atsuko Ota, Hiroaki Kitamura, Keigo Sugimoto, Miho Ogawa, Naoshi Dohmae, Hiroki Okuno, Kazuya Takahashi, Kazutaka Ikeda, Tsutomu Tomita, Naoki Matsuoka, Kunitaka Matsuishi, Tetsuro Inokuma, Tohru Nagano, Makoto Takeo, Takashi Tsuji

**Affiliations:** 1 Aderans Company Limited, Shinjuku, Tokyo, Japan; 2 OrganTech Inc., Chuo-ku, Tokyo, Japan; 3 Laboratory for Organ Regeneration, RIKEN Center for Biosystems Dynamics Research (BDR), Kobe, Hyogo, Japan; 4 Biomolecular Characterization Unit, RIKEN Center for Sustainable Resource Science, Wako, Saitama, Japan; 5 RIKEN, Nishina Center for Accelerator-Based Science, Wako, Saitama, Japan; 6 Department of Applied Genomics, Laboratory of Biomolecule Analysis, Kazusa DNA Research Institute, Kisarazu, Chiba, Japan; 7 Biobank, National Cerebral and Cardiovascular Center, Suita, Osaka, Japan; 8 Kobe City Medical Center General Hospital, Kobe, Hyogo, Japan; University of Delhi, INDIA

## Abstract

Globally, the rapid aging of the population is predicted to become even more severe in the second half of the 21st century. Thus, it is expected to establish a growing expectation for innovative, non-invasive health indicators and diagnostic methods to support disease prevention, care, and health promotion efforts. In this study, we aimed to establish a new health index and disease diagnosis method by analyzing the minerals and free amino acid components contained in hair shaft. We first evaluated the range of these components in healthy humans and then conducted a comparative analysis of these components in subjects with diabetes, hypertension, androgenetic alopecia, major depressive disorder, Alzheimer’s disease, and stroke. In the statistical analysis, we first used a student’s *t* test to compare the hair components of healthy people and those of patients with various diseases. However, many minerals and free amino acids showed significant differences in all diseases, because the sample size of the healthy group was very large compared to the sample size of the disease group. Therefore, we attempted a comparative analysis based on effect size, which is not affected by differences in sample size. As a result, we were able to narrow down the minerals and free amino acids for all diseases compared to *t* test analysis. For diabetes, the *t* test narrowed down the minerals to 15, whereas the effect size measurement narrowed it down to 3 (Cr, Mn, and Hg). For free amino acids, the *t* test narrowed it down to 15 minerals. By measuring the effect size, we were able to narrow it down to 7 (Gly, His, Lys, Pro, Ser, Thr, and Val). It is also possible to narrow down the minerals and free amino acids in other diseases, and to identify potential health indicators and disease-related components by using effect size.

## Introduction

Globally, the rapid aging of the population is expected to accelerate in the latter half of the 21st century. Under these circumstances, disease prevention, care, and health promotion efforts will be important, especially in the developed countries where the largest shifts are expected to take place [1]. Regular health checkups play an essential role in disease prevention and are beneficial for early detection and treatment [2]. In particular, they are expected to facilitate specific behavioral modifications to assist individuals in extending their healthy life expectancy through lifestyle awareness in youth and middle age and through improved awareness of health management. Currently, biomarkers or risk markers in human body fluids, such as blood and urine, are commonly used to diagnose various diseases and for health checkups [3,4]. Furthermore, researchers worldwide are actively working to identify new disease markers, such as blood markers, for cancer and Alzheimer’s disease (AD) [3,5]. However, diagnostic procedures using these body fluids may be affected by dietary factors or by exercise performed immediately before the checkup; in addition, these values show considerable daily fluctuations. Patients need to visit a hospital or health checkup center to undergo a noninvasive urine test, and blood-based diagnostic procedures require an invasive step. To address this issue and extend healthy life expectancy in the aging society of the near future, there is a demand for the establishment of a convenient and stable diagnostic procedure.

Hair shafts have a variety of types, including head, body, and pubic hair [6]. A hair shaft is produced by a hair follicle, one of the appendices of skin system [6]. To produce hair shafts, hair matrix cells in the hair bulb divide and are then fibrillated by the addition of keratins; through this process, hair grows at a rate of 1 cm per month [6]. Hair shaft lengths are determined by the hair cycle, in which the follicle passes through the stages of anagen, catagen, and telogen, regenerating with each cycle [7]. The hair follicle is the only organ known to undergo full regeneration in adults. The head hair shafts, for which the hair cycle is estimated to be approximately 3–7 years, are unique among the tissues of the living body in that they retain a long physical “archive” as they grow [8,9]. Hair matrix cells are known to selectively accumulate minerals, amino acids, steroid hormones, lipids, and metabolites through their cell membranes from the bloodstream [9]. These components remain in the hair shaft, which effectively immobilizes them [9]. For this reason, the hair shaft can be used for site-specific data acquisition and is considered a more stable source of information than body fluids, which show daily fluctuations caused by cell membrane selectivity and cell homeostasis [9]. This unique characteristic of hair has been applied to several fields; for example, drug metabolites are site-specifically measured with high sensitivity to provide evidence in the field of forensic science, and hair samples are used to detect excess ingestion of dangerous heavy metals such as Hg and arsenic (As) in the field of environmental science [10,11]. Based on the above findings, the hair shaft is considered to be the only biological sample or cellular specimen that provides a site-specific archive with very few intraday and daily fluctuations, unlike body fluids [9]. Therefore, if laboratory tests using the hair shaft as a sample can be realized, they can serve as noninvasive examinations that do not necessitate a clinic visit and will greatly contribute to the understanding of health indices, the accuracy of diagnosis, and the extension of healthy life expectancy [9].

Several studies have reported on the feasibility of disease diagnosis using head hair samples and assayed minerals and constituent amino acids contained in the samples [12,13]. Correlations between hair mineral composition and various diseases, such as hypertension (HT) [13,14], diabetes mellitus (DM) [12,15], and heart diseases [16,17], and mental disorders [18,19] have been demonstrated by previous studies. Regarding amino acids contained in proteins, concentrations of the nonessential amino acids Gly and Glu and the essential amino acid Ile in head hair samples are reported to be higher in patients with type II diabetes mellitus than in healthy volunteers [20]. However, the presence of free-amino acids in hair samples have not been reported. Previous studies have analyzed the raw data obtained from patients with diseases and from healthy subjects and determined the differences using statistical significance tests, such as Student’s *t* test [21]. However, these have been controversial because influencing factors cannot be narrowed down owing to the presence of several factors involved in disease, and inconsistencies between data have been identified and have occurred as result of discrepancies in pretreatment conditions in the reported studies [21]. Nevertheless, head hair diagnostics have the potential to serve as a useful diagnostic tool for the early detection of health indicators and diseases in that they are noninvasive, have very few daily fluctuations and take advantage of a historical “archive” [9].

In this study, we compared the mineral and free amino acid components under consistent pretreatment conditions for assays between healthy subjects and patients with six different conditions causing changes in head hair patterns, namely, DM, HT, androgenetic alopecia (AGA), major depressive disorder (MDD), AD and cerebral infarction (CI), in an attempt to identify an illness-related marker. In general, the healthy subjects considered to have no apparent abnormalities (NAA) in their health checkup [22]. However, we examined statistical variations in the healthy subjects because there may be variations from healthy to disease onset, and variations in eating and drinking habits owing to environmental and regional differences were considered. We performed a statistical analysis using Student’s *t* test of the healthy subjects and patient groups to compare mineral components with those reported by previous studies. Furthermore, we conducted a statistical analysis of free amino acids in the head hair shafts, which have not been sufficiently analyzed in previous studies. Furthermore, although sample size inherently affects two-group comparisons, we attempted to examine effect sizes such that two- group comparisons could be quantified without being affected by sample size, thereby narrowing the range of disease-related components to a greater extent than conventional statistical analyses.

## Materials and methods

### Subjects

The subjects included in this study were divided into two groups: the healthy subjects (healthy group) and the patient subject group (patient group). The healthy group included 1,095 residents (age21–82) of Japan aged >20 years who provided informed consent in advance in response to the internet recruitment of volunteers for this study. The patient group included 124 residents (age26–88) of Japan aged >20 years who regularly visited a doctor to treat DM, HT, AGA, MDD, AD, or CI (including patients with two or more of these diseases) and provided informed consent in advance with the cooperation of the affiliated medical institutions. This study was conducted after obtaining written consent for clinical research from Japan Conference of Clinical Research (Approval number: 258). The participants were recruited to the study from June 27, 2019. The human subjects’ data/samples were collected from June 27, 2019 to February 6, 2020. We conducted this study from April 1, 2017. Only from a specific PC in the data management room, authors had access to information that could identify individual participants during or after data collection.

### Methods of disease diagnosis

For DM, diagnosis was conducted through blood glucose level and HbA1c tests by physicians at the hospital. HT was diagnosed by physicians at hospitals in Japan. Additionally, out of 45 cases, 20 were referred to the National Cerebral and Cardiovascular Center from other hospitals for further examination and treatment. AGA was diagnosed through interviews and visual assessments by a dermatologist at the Kobe City Medical Center General Hospital. MDD and AD were diagnosed according to the DSM-5 diagnostic criteria by psychiatric and neurological specialists at the Kobe City Medical Center General Hospital. CI was diagnosed through imaging tests at the National Cerebral and Cardiovascular Center.

### Sample collection and pretreatment of head hairs

After approval by the Human Ethics Committee (Approval number: 258) and informed consent from the donors, head hair shafts were collected by cutting hairs at their roots on the back of the head with a dedicated head hair collection kit. The head hairs were cut 3 cm from their roots, and dirt on the surfaces was wiped away with an alcohol swab. Eight milligrams of clean head hairs were placed, along with metal cones (MC-0316(S), Yasui Kikai Corporation), into a freeze-fracture tube (ST-0320PCF, Yasui Kikai Corporation) and loaded onto an aluminum block. The aluminum block with the mounted freeze-fracture tube was cooled in a container with liquid nitrogen and fractured in a multi-sample cell disruptor (Multi-beads shocker, Yasui Kikai Corporation). After fracture, the sample was incubated until the freeze-fracture tube returned to room temperature. Milli-Q water was added to the disrupted samples to prepare a 12 μg/μL suspension, and the metal cones were removed from the tube. The fractured head hairs were dispersed well in Milli-Q water, and 100 μL of suspension (containing 1.2 mg of head hairs) was dispensed into individual tubes for analysis.

### Mineral analysis

A mixture of nitric acid and perchloric acid in a ratio of 3:1 was added to the head hair suspension and heated in a closed container at 60–70°C for 1.5 hours. It was ensured that all hair residues had dissolved, and a constant volume was prepared with ultrapure water. An ICP-MS assay (NexION 300 Mass Spectrometer, PerkinElmer Japan) was performed.

### Free amino acid assay

Free amino acids dispersed in water were incubated for one night or more. The samples were centrifuged to collect the supernatant. Then, 25 μL of borate buffer, 5 μL of n-valine, 10 μL of the collected sample, and 10 μL of AQC were mixed, sealed, and heated in a heat block at 55°C for 10 min. An HPLC apparatus (Agilent 1200 series, Agilent Technologies, Inc., Santa Clara, CA, USA) equipped with a diode array detector (G1315C) was used to measure free amino acids [23].

### Analysis methods

A significance test was performed to compare the measured values obtained by the analysis methods from the healthy and patient groups. Student’s *t* test was used as the significance test, with the significance threshold set to 0.05. The effect sizes were also used for analysis. To calculate the effect sizes, Cohen’s *d* was used, and the components with effect sizes of 0.8 or greater were assumed to be important factors.

## Results

### Analysis of variations among various components in the healthy subjects

**Fig 1:**
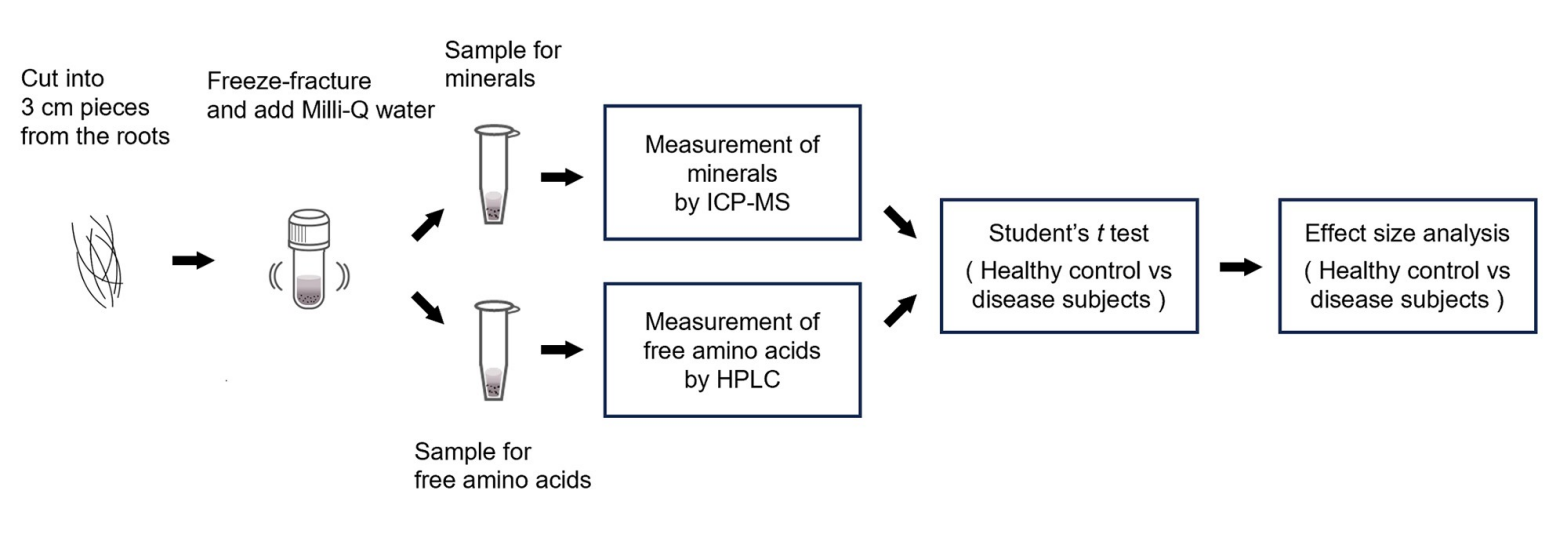
The scheme showing the methods in this study. Hair was cut into 3 cm length pieces from the root, frozen and crushed using a metal cone. After crushing, Milli-Q water was added, the metal cone was removed, and the dispersion liquid was divided into one for mineral measurement and one for free amino acid measurement. Minerals in hair were measured by ICP-MS, and free amino acids were measured by HPLC. The measured values of the healthy controls and subjects with diseased were compared using *t* tests and effect sizes.

Hair was cut into 3 cm length pieces from the root, crushed by freeze-fracturing, and Milli-Q water was added to the tube containing crushed hair. Minerals in hair were measured by ICP-MS and free amino acids by HPLC, and measurements from healthy controls and diseased subjects were compared using *t* tests and effect sizes (Fig 1). Most previous studies defined the term “healthy subjects” as those with no apparent abnormalities in medical diagnoses such as health checkups [12]. However, it is possible that those “healthy subjects” may include people with any presymptomatic disease or those unaware of the presence of a disease in addition to healthy subjects with no disease whatsoever. Therefore, it is necessary to clarify the variations in hair components in healthy subjects to address this issue and establish diagnostic procedures using head hair for health and illness diagnosis. In this study, we used a questionnaire survey to obtain information on health conditions and lifestyles. First, we defined 1,095 volunteers having no subjective symptoms or having received no illness diagnosis as healthy subjects. Then, we collected head hair samples to analyze the minerals and free amino acids in the samples according to the method as described in the Materials and Methods. The measured values of the individual components in the head hair samples were converted into values per 1 mg head hair. The distribution of the converted values was analyzed using a box-and-whisker diagram, and outliers of ≥5% were selected as components with large variations.

**Fig 2:**
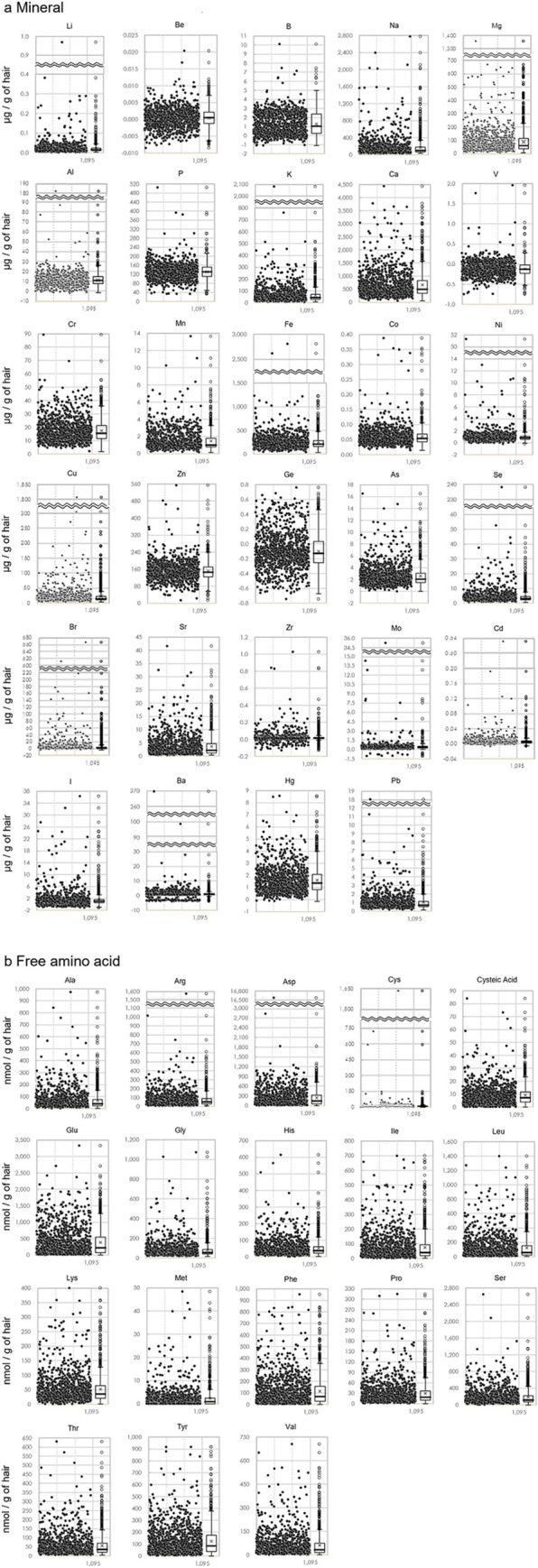
Box-and-whisker plot showing the content of the components of interest in the head hair samples from the healthy group. Box-and-whisker plots showing (a) the mineral contents and (b) the free amino acid contents in the head hair samples from the healthy group.

ICP-MS was used for mineral components to detect all 110 elements simultaneously. An assay performed on 29 types of elements above the detection limits revealed the presence of several outliers in Li, Na, Mg, K, Ca, Mn, Fe, Ni, Cu, As, Se, Br, Sr, Zr, Cd, I, Ba and Pb (Fig 2A, Table 1). Amino acids were classified into those composing proteins and free amino acids. In the present study, free amino acids were measured in head hair samples because free amino acids are used in determining risks for illnesses using blood samples. We analyzed eight types of essential amino acids; nine types of nonessential amino acids; and cysteic acid, a unique component in head hairs. Our outcomes demonstrated that several outliers were identified in nearly all amino acids except cysteic acid and tyrosine (Fig 2B, Table 1). Many statistical outliers were noted in the distributions of minerals and free amino acid components among healthy subjects. The components in which individual differences were observed, i.e., the components showing outliers, have a high potential to serve as biomarkers suggesting the effects of illness and/or differences in food and drink in terms of environmental science.

**Table 1:** Components with ≥0.5% outliers in the head hair samples from the healthy group.

Minerals	Free amino acids
Li/Na/Mg/K/Ca/Mn/Fe/Ni/Cu/ As/Se/Br/ Sr/Zr/Cd/I/Ba/Pb	all except cysteic acid/Tyr

The table shows the minerals and free amino acids with ≥0.5% outliers in the head hair samples from the healthy group.

### Comparison of various types of components in head hair samples between the healthy subjects and patient

**Fig 3:**
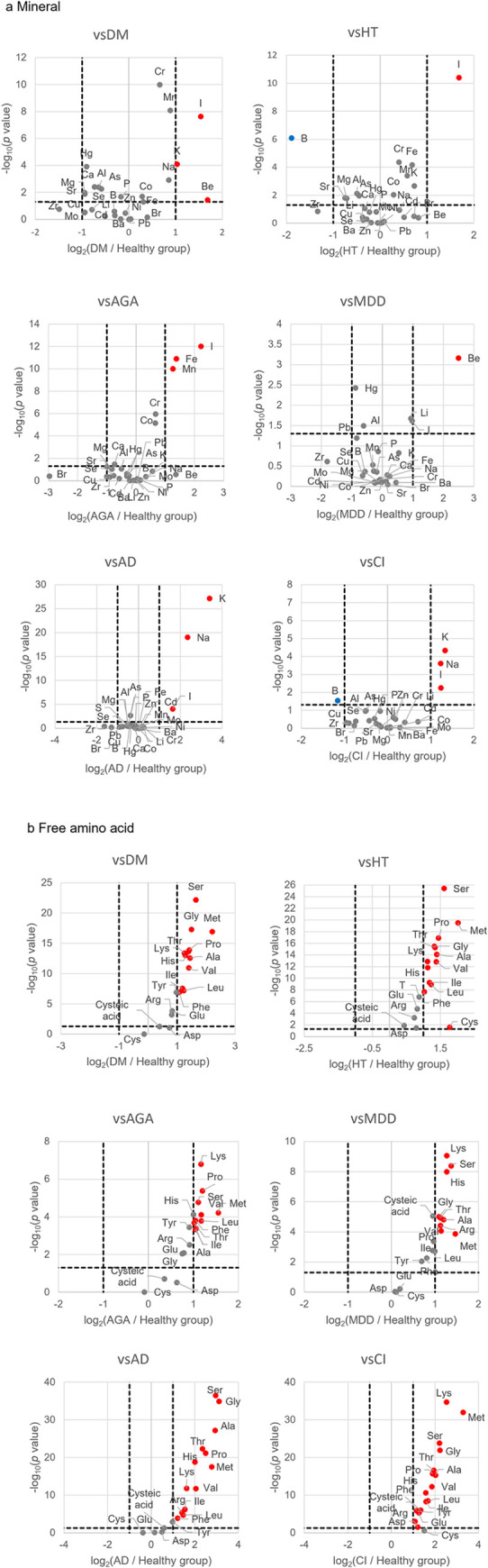
A volcano plot created from the measurement values of the healthy group and the disease group. (a) Volcano plots created from the mineral content in head hair samples of the healthy group and the disease group, and (b) volcano plots created from free amino acid content in head hair samples of the healthy group and the disease group. Red plot represents high expression, and blue plot represents low expression.

We compared healthy subjects and patients with various diseases in this study to identify a new diagnostic marker for illness. The examined illnesses included DM (n = 34, age 31–88), HT (n = 45, age 27–88), AGA (n = 18, age 26–61), MDD (n = 20, age 38–75), AD (n = 13, age 67–88), and CI (n = 15, age 46–87). A comparison of minerals and the free amino acids component contents in head hair samples was performed between the healthy subjects and patients with each of the six diseases. The results were analyzed via the conventionally used Student’s *t* test. Differences in the contents of 29 types of minerals in the head hair samples were evaluated between the healthy subjects and patients with six diseases (Tables 2 and S1). A volcano plot was created from the obtained *p* values and measurement values, and the results shown by the volcano plot and the *t* test were consistent. (Fig 3). We did not conduct age and result correlation analysis this time.

**Table 2:** Components with statistically significant differences observed between the healthy and patient groups by Student’s *t* test.

Disease	Minerals	Free amino acids
DM	Be/Na/Mg/Al/P/K/Ca/Cr/ Mn/Fe/Co/As/Sr/I/Hg	all except Asp/Cys/cysteic acid
HT	B/Mg/Al/K/Cr/Mn/ Fe/Co/As/Sr/I	all
AGA	Ca/Cr/Mn/Fe/Co/I	all except Asp/Cys/cysteic acid
MDD	Li/Be/Al/I/Hg	all except Asp/Cys/Glu
AD	Na/P/K/I	all except Asp/Cys/Glu
CI	B/Na/K/I	all except Cys

A comparison between the healthy and patient groups by Student’s *t* test identified minerals and free amino acids with *p* values < 0.05, as shown in the table.

The results demonstrated that statistically significant differences in Be, Na, Mg, Al, P, K, Ca, Cr, Mn, Fe, Co, As, Sr, I and Hg were observed in the subgroup of patients with DM; those in B, Mg, Al, K, Cr, Mn, Fe, Co, As, Sr and I in the subgroup of patients with HT; those in Ca, Cr, Mn, Fe, Co and I in the subgroup of patients with AGA; those in Li, Be, Al, I and Hg in the subgroup of patients with MDD; those in Na, P, K and I in the subgroup of patients with AD; and those in B, Na, K and I in the subgroup of patients with CI. Differences in the contents of 18 types of free amino acids in the head hair samples were evaluated between the healthy and patient subjects with six diseases (Tables 2 and S2). We observed significant differences in the free amino acids other than Asp, Cys and cysteic acid in the subgroup of patients with DM; those in all the amino acids in the subgroup of patients with HT; those in the free amino acids other than Asp, Cys and cysteic acid in the subgroup of patients with AGA; those in the free amino acids other than Asp, Cys and Glu in the subgroup of patients with MDD; those in the free amino acids other than Asp, Cys and Glu in the subgroup of patients with AD; and those in the free amino acids other than Cys in the subgroup of patients with CI. As shown in Table 2, the results of Student’s *t* test clarified that statistically significant differences were observed in many components of minerals and free amino acids.

### Statistical analysis of the effect sizes of various components in the head hair samples obtained from healthy subjects and patient groups

The results of the *t* test in previous studies showed significant differences in numerous components between the healthy and patient groups. Therefore, we analyzed the effect sizes between the healthy and patient groups to narrow down the list of candidates for illness markers. The effect size was considered to have a statistical advantage, owing to which the results of the intergroup comparisons could be quantified without an effect of their sample sizes [24]. In the present study, components with an effect size of ≥0.8 were considered to differ between healthy and patient groups.

The contents of 29 minerals in the head hair samples were examined in the healthy and patient groups (Tables 3 and S3). Differences were observed in Cr, Mn and Hg in the subgroup of patients with DM, Cr and Co in patients with AGA, and P in patients with AD. Additionally, the contents of 18 free amino acids in the head hair samples were examined in the healthy and patient groups (Tables 3 and S4). Differences in Gly, His, Lys, Pro, Ser, Thr and Val were observed in the subgroup of patients with DM; those in Lys, Met and Ser in the subgroup of patients with HT; those in Lys and Pro in the subgroup of patients with AGA; those in Lys in the subgroup of patients with AD; and those in Glu, Ile, Leu, Phe, Pro, Tyr and Val in the subgroup of patients with CI. The contents of minerals and free amino acids were compared between healthy and patient subgroups based on the effect size. The results were consistent with the components that were significantly different by the *t* test and demonstrated that this analysis can reduce the number of candidate disease-specific components. Therefore, our outcomes suggested that the list of potential disease-related components may have been narrowed down.

**Table 3:** Components considered important on the basis of effect size.

Disease	Minerals	Free amino acids
DM	Cr/Mn/Hg	Gly/His/Lys/ Pro/Ser/Thr/Val
HT	none	Lys/Met/Ser
AGA	Cr/Co	Lys/Pro
MDD	none	Lys
AD	P	His/Lys
CI	none	Glu/Ile/Leu/Phe/Pro/Tyr/Val

An analysis based on the effect sizes for the comparisons between the healthy and patient groups identified several minerals and free amino acids with Cohen’s *d* values of ≥0.8, as shown in the table.

## Discussion

**Fig 4:**
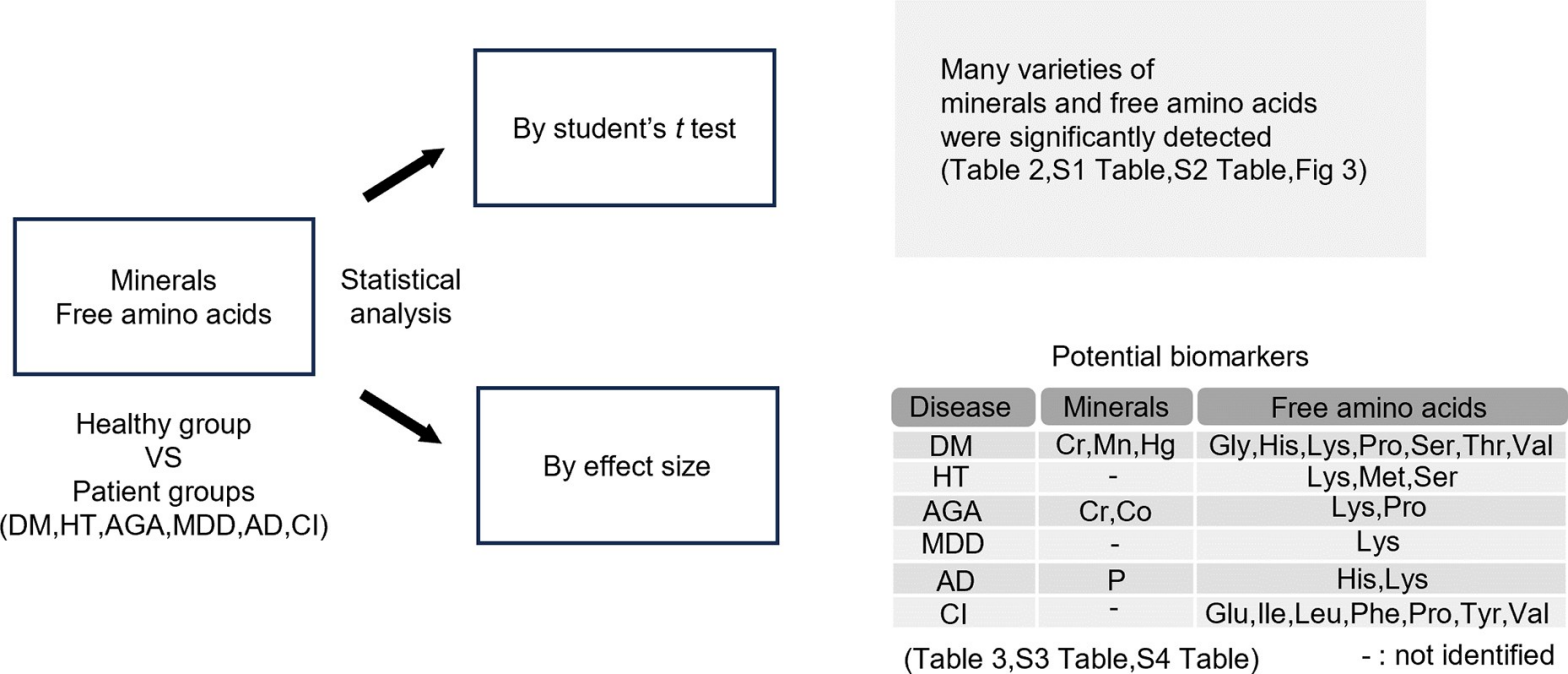
Statistical analysis methods used in this study and results obtained from these analyses. The measurement data of minerals and free amino acids contained in the hair of healthy subjects and patient groups with diseases (DM, HT, AGA, MDD, AD, CI) was analyzed using Student’s *t* test and effect size, and the results obtained from these analyses are shown.

In the present study, we aimed to establish a new health indicator or disease diagnosis method based on mineral and free amino acid components in hair. After confirming the variation of various components in healthy subjects. We subsequently analyzed the effect size as a statistical method in addition to the significance difference test by *t* test. As a result, many variations in the various components were observed even in healthy subjects. Statistical analysis of the various components in the hair of healthy subjects and diseased patients revealed that the analysis of effect sizes was even more effective than the *t* test in narrowing down candidate components (Fig 4). These findings indicated the need to clarify the definitions of healthy people and patients with diseases and the possibility of narrowing down health indicators and disease-related components through appropriate statistical analysis.

Previous studies have typically defined a healthy person as one without abnormalities from clinical examination of the disease [12,22]. In this case, one would expect to include both genuinely healthy individuals and those with near-abnormal clinical laboratory results. However, in patients with a disease, when treatment is initiated after clinical tests show abnormal values, the abnormal values decrease and approach normal values. Therefore, the strict definition of the disease component, both in healthy subjects and in patients with the disease, will significantly impact the identification of the disease component. Few papers have reported on the variation in the number of mineral components in the hair of healthy individuals on a large scale [21]. In addition to the effects of a disease, they are known to be affected by environmental scientific influences, i.e., the accumulation of toxic substances from the environment and drinking water and food from the living environment [10,25]. Hence, the mineral components that are considered to be susceptible to these factors must be distinguished from the elements not susceptible to them in homeostasis in the living body, e.g., the Na/K balance, which is ongoing [26]. In the present study, we discussed the detectable components of minerals and free amino acids in hair samples from healthy subjects, who self-reported as NAA, via box-and whisker analysis similar to previous studies on the subject [27]. The results suggested that the components were divided into two types: those with a wider interquartile range and those with a narrow interquartile range. The former components were strongly associated with environmental factors, dietary lifestyle, and illnesses. Ca is known to be related to water quality [28], while As and Hg seem to be associated with environmental factors such as soil and water quality [29,30]. Previous studies have shown correlations between arsenic concentrations in fish and human hair in arsenic-contaminated and uncontaminated areas, as well as concerns about the bioaccumulation of heavy metals in fish in the food chain and the intake of heavy metals in the body through diet [31]. Taking these studies into account, it is conceivable that the measured values of minerals and free amino acids in this study may be influenced by changes related to dietary habits and environmental factors. Therefore, there is considerable interest in the potential for these measurements to serve as biomarkers not only for diseases but also for dietary and environmental sciences. In addition, many minerals and free amino acids are 5% outliers in the box-and-whisker statistical analysis, suggesting that there may be healthy subjects whose values have not reached abnormal values in the laboratory tests or disease-related components in these, along with environmental factors and diet. Therefore, to more accurately identify disease-related components, it will be necessary to clarify the definition of “diseased patients” by eliminating environmental factors in healthy individuals, distinguishing elements such as Na and K, whose amounts are regulated by cell biology, and by having healthy individuals and disease markers at or above a certain value.

The *t* test was the conventional statistical method for identifying a disease marker among various components in the hair samples [21]. Many studies have reported the components for which significant differences in contents were found in the hair samples from both the healthy and patient groups by the *t* test [32–34]. In a previous paper, owing to difficulty in narrowing down markers via *t* test alone, additional analysis was attempted for marker identification by evaluating the correlation coefficient between two of the components in hair between healthy subjects and diseased patients and the component loadings of each component in hair when PCA was performed on the components in hair [32]. In addition to the evaluation of individual components, reports have demonstrated the presence of significant differences in the ratio of two components between healthy and patient groups for pairs of components that interact or antagonize each other *in vivo* (e.g., Cu and Zn) [35,36]. However, one of the merits of the effect size is that the results of the intergroup comparison can be quantified without being influenced by their sample sizes [24]. The present study was compared with previous reports on mineral components in hair that showed significant differences by Student’s *t* test between healthy and patient groups. Then, in patients with DM, seven elements (Na, Mg, K, Ca, Cr, Mn and As) out of the 15 elements showed significant differences in this study, and in patients with HT, five elements (K, Cr, Mn, Fe and Co) out of the 11 elements that showed significant differences in this study were consistent [21]. Further analysis by effect size narrowed down Cr, Mn and Hg as important factors in DM (of these, Cr and Mn were consistent with previous reports) but not the important elements in HT. The comparison of free amino acid amounts by *t* tests showed significant differences in all amino acids except His, Thr and Val in patients with DM and all amino acids in patients with HT. Analysis by effect size successfully narrowed down Gly, His, Lys, Pro, Ser, Thr and Val for DM and Lys, Met and Ser for HT as important factors. Thus, this study demonstrated the potential for more effectively narrowing down candidate disease markers using effect size analysis.

To date, in the field of blood analysis, there have been several reports on biomarkers of amino acids and their association with nutritional status, liver disease, and cancer [37]. The free amino acids used for protein and hormone synthesis are important elements reflecting *in vivo* conditions. Foods are degraded into free amino acids and peptides *in vivo*, absorbed from the small intestine epithelial mucosa, and supplied to the liver via the bloodstream. In the liver, it was reported that approximately 2,000 enzymes initiate 500 different types of chemical reactions instantaneously, during which 600,000–1,000,000 proteins are produced from a single hepatic cell per minute. Therefore, the association of amino acids with liver diseases has been frequently reported. As already known, the plasma free amino acid profile is subject to the influence of specific illnesses, including cancers; the plasma free amino acid profiles specific to cancer patients have been shown [38,39]. However, few reports exist on free amino acid markers in hair; accordingly, we searched for disease-specific free amino acids in hair in this study. In the present study, analysis of free amino acids in the hair of patients with disease narrowed down Gly, His, Lys, Pro, Ser, Thr and Val as important factors in the analysis of effect sizes in patients with DM. This result is not entirely consistent with previous studies showing that Gly, Glu and Ile are DM markers in hair, suggesting that a clear definition of healthy and diseased hair and a more detailed analysis are needed. Another report stated that amino acids facilitate disease determination by analysis of the overall balance rather than of specific markers [40]. Although this study succeeded in narrowing down the important factors, a future analysis focusing on blood amino acids and the balance of amino acids may also be important.

Since hair, which is a specimen of immobilized cells, retains the body’s intracellular information for a long time, it is a biological sample that has potential for application to individual health indicators and disease diagnoses. Furthermore, since the area of the head hair can identify the period, it is possible to measure the transition to disease and the degree of recovery after treatment, which may be useful for disease diagnosis and prognosis. In other words, head hair diagnosis is expected to become an effective diagnostic method for health indicators and disease diagnosis for many people, with usefulness that differs from urine and blood, which have high daily fluctuations. Toward the realization of this diagnostic method, the present study analyzed free amino acids in head hair, which have not been fully reported thus far, and minerals were analyzed as well. This study demonstrated the possibility of narrowing down a list of possibly disease-related components by statistical analysis using effect sizes. For each disease, diagnosis was conducted by physicians at specialized hospitals based on examination data, and we conducted our analysis using the acquired data. Information other than diagnosis, such as medication and disease severity, was not collected in this study. Furthermore, factors such as participant’s dietary habits, environment, and aging can affect both systemic and peripheral metabolism, so considering these factors is a task for future research. However, even so, the results of this study show the differences in minerals and free amino acids present in the hair of healthy individuals and patients with diseases, providing valuable insights. In future studies, we will investigate clearly defining healthy individuals and the level of disease, such as immediately after the onset of disease, and identifying disease-specific markers by raising the comparative quantities and considering appropriate statistical processing after making the differences in the internal conditions of healthy individuals and patients with the disease more pronounced. In addition, we hope to conduct a prospective cohort study on various illnesses to correlation data with blood and analyze time-series data to reveal the causal relationship between *in vivo* events at the onset of an illness and the components in the head hair samples.

## Supporting information

S1 Table Means ± SDs for the individual minerals and the *p* values between the healthy and patient groups. (PDF)

S2 Table Means ± SDs for the individual free amino acids and the *p* values between the healthy and patient groups. (PDF)

S3 Table A comparison of the effect sizes of minerals between the healthy and patient groups. (PDF)

S4 Table A comparison of the effect sizes of free amino acids between the healthy and patient groups. (PDF)

S5 Table Raw data of hair shaft components measurements for healthy control and each diseased subject. (XLSX)

S1 Dataset (XLSX)
