# Supplementary material for: Comparative studies of hair shaft components between healthy and diseased donors
Source: PLoS One. 2024 May 8;19(5):e0301092. doi: 10.1371/journal.pone.0301092 (PMC11078425; doi:10.1371/journal.pone.0301092)
Supplement: S1 Table — (PDF) [file pone.0301092.s001.pdf]

S1 Table Means ± SDs for the individual minerals and the *p* values between the healthy and patient groups

	HC	DM		HT		AGA		MDD		AD		CI	
	Mean ± SD	Mean ± SD	*p* value	Mean ± SD	*p* value	Mean ± SD	*p* value	Mean ± SD	*p* value	Mean ± SD	*p* value	Mean ± SD	*p* value
Li	0.02 ± 0.04	0.02 ± 0.02	0.517	0.02 ± 0.01	0.364	0.02 ± 0.02	0.886	0.05 ± 0.09	**<0.05**	0.03 ± 0.01	0.679	0.03 ± 0.01	0.470
Be	0.00 ± 0.00	0 ± 0	**<0.05**	0.00 ± 0.00	0.406	0.00 ± 0.00	0.271	0.00 ± 0.00	**<0.001**	N.D.	N.D.	N.D.	N.D.
B	1.31 ± 1.28	1.06 ± 0.8	0.246	0.35 ± 1.16	**<0.001**	1.05 ± 0.57	0.385	1.08 ± 0.58	0.416	0.98 ± 1.00	0.350	0.59 ± 0.44	**<0.05**
Na	175.75 ± 237.85	317.58 ± 521.04	**<0.01**	233.23 ± 277.27	0.115	221.32 ± 332.04	0.424	206.73 ± 325.36	0.567	892.02 ± 1383.64	**<0.001**	410.96 ± 592.61	**<0.001**
Mg	91.21 ± 99.25	47.28 ± 37.3	**<0.05**	55.48 ± 42.89	**<0.05**	51.22 ± 21.12	0.088	84.60 ± 60.57	0.767	65.73 ± 52.94	0.356	79.46 ± 91.05	0.648
Al	11.88 ± 8.65	7.6 ± 3.11	**<0.01**	8.41 ± 4.64	**<0.01**	8.36 ± 2.40	0.085	7.72 ± 3.51	**<0.05**	8.24 ± 4.58	0.130	8.31 ± 3.75	0.111
P	132.37 ± 36.86	117.57 ± 25.73	**<0.05**	124.37 ± 30.39	0.151	136.08 ± 23.16	0.670	120.00 ± 32.61	0.137	100.97 ± 16.92	**<0.01**	117.10 ± 34.39	0.111
K	66.36 ± 89.33	135.69 ± 284.43	**<0.001**	109.67 ± 154.23	**<0.01**	97.51 ± 88.44	0.143	96.16 ± 167.71	0.148	701.10 ± 1742.27	**<0.001**	166.81 ± 283.26	**<0.001**
Ca	673.55 ± 535.18	404.9 ± 383.41	**<0.01**	535.18 ± 462.80	0.088	404.55 ± 177.47	**<0.05**	751.30 ± 609.67	0.521	581.69 ± 445.76	0.538	593.66 ± 585.62	0.566
V	N.D.	N.D.	N.D.	N.D.	N.D.	N.D.	N.D.	N.D.	N.D.	N.D.	N.D.	N.D.	N.D.
Cr	17.52 ± 8.83	27.7 ± 12.46	**<0.001**	23.08 ± 10.40	**<0.001**	27.81 ± 8.08	**<0.001**	16.68 ± 9.93	0.672	19.81 ± 8.40	0.352	19.86 ± 12.33	0.311
Mn	1.47 ± 1.22	2.72 ± 1.7	**<0.001**	2.20 ± 3.09	**<0.001**	3.53 ± 4.39	**<0.001**	1.19 ± 1.26	0.295	1.52 ± 1.33	0.904	1.50 ± 1.49	0.940
Fe	251.51 ± 178.03	312.33 ± 155.31	**<0.05**	401.85 ± 889.00	**<0.001**	651.83 ± 1384.99	**<0.001**	235.39 ± 147.07	0.688	251.73 ± 97.87	0.997	261.37 ± 210.21	0.832
Co	0.06 ± 0.03	0.07 ± 0.02	**<0.05**	0.07 ± 0.04	**<0.01**	0.09 ± 0.05	**<0.001**	0.06 ± 0.02	0.707	0.06 ± 0.02	0.963	0.06 ± 0.02	0.925
Ni	1.05 ± 1.83	1.08 ± 0.77	0.924	1.08 ± 0.69	0.904	1.07 ± 0.46	0.965	0.93 ± 0.33	0.763	1.23 ± 1.17	0.728	0.94 ± 0.38	0.822
Cu	22.27 ± 59.03	11.6 ± 8.07	0.292	17.27 ± 20.08	0.571	11.08 ± 5.22	0.422	14.19 ± 7.09	0.541	11.78 ± 8.53	0.522	11.43 ± 8.82	0.477
Zn	150.25 ± 46.62	142.39 ± 27.74	0.329	150.56 ± 29.60	0.964	148.70 ± 21.01	0.888	147.44 ± 45.64	0.789	134.92 ± 23.81	0.237	164.15 ± 36.48	0.251
Ge	N.D.	N.D.	N.D.	N.D.	N.D.	N.D.	N.D.	N.D.	N.D.	N.D.	N.D.	N.D.	N.D.
As	2.57 ± 1.78	1.71 ± 0.87	**<0.01**	1.88 ± 1.44	**<0.05**	2.18 ± 1.83	0.352	2.26 ± 1.40	0.437	1.84 ± 0.77	0.141	1.82 ± 1.22	0.102
Se	4.52 ± 8.36	2.6 ± 1.42	0.183	3.69 ± 3.73	0.507	2.64 ± 0.97	0.342	3.04 ± 1.29	0.429	2.63 ± 1.53	0.416	2.71 ± 1.67	0.402
Br	7.56 ± 31.83	9.94 ± 33.87	0.669	12.47 ± 39.33	0.316	0.97 ± 3.14	0.380	8.51 ± 17.31	0.895	3.08 ± 4.03	0.612	4.43 ± 4.49	0.703
Sr	3.66 ± 4.09	1.9 ± 2.02	**<0.05**	2.17 ± 2.34	**<0.05**	1.82 ± 1.07	0.058	3.97 ± 3.34	0.735	2.53 ± 2.10	0.323	2.72 ± 3.73	0.375
Zr	0.02 ± 0.06	0.01 ± 0.01	0.171	0.01 ± 0.01	0.140	0.01 ± 0.01	0.470	0.01 ± 0.01	0.243	0.01 ± 0.01	0.372	0.01 ± 0.01	0.513
Mo	0.48 ± 1.3	0.25 ± 0.16	0.303	0.46 ± 0.60	0.929	0.53 ± 0.94	0.873	0.40 ± 0.16	0.781	0.43 ± 0.19	0.901	0.50 ± 0.28	0.953
Cd	0.01 ± 0.02	0.01 ± 0	0.568	0.01 ± 0.03	0.365	0.00 ± 0.00	0.643	0.01 ± 0.00	0.819	0.01 ± 0.00	0.940	0.01 ± 0.01	0.426
I	1.61 ± 2.99	4.68 ± 6.19	**<0.001**	5.16 ± 9.66	**<0.001**	7.51 ± 13.94	**<0.001**	3.16 ± 4.87	**<0.05**	5.01 ± 8.22	**<0.001**	3.78 ± 3.97	**<0.01**
Ba	1.4 ± 8.88	1.24 ± 0.86	0.917	1.22 ± 0.91	0.894	1.20 ± 0.48	0.926	1.90 ± 1.12	0.800	1.47 ± 0.74	0.977	1.70 ± 1.68	0.895
Hg	1.6 ± 1.12	0.86 ± 0.57	**<0.001**	1.36 ± 1.06	0.160	1.29 ± 0.91	0.237	0.87 ± 0.81	**<0.01**	1.26 ± 1.22	0.274	1.30 ± 0.65	0.294
Pb	1.05 ± 1.12	1.05 ± 0.8	0.995	1.11 ± 0.72	0.728	1.09 ± 0.80	0.880	0.59 ± 0.22	0.064	0.77 ± 0.47	0.355	0.87 ± 0.50	0.538
