# Supplementary material for: Comparative studies of hair shaft components between healthy and diseased donors
Source: PLoS One. 2024 May 8;19(5):e0301092. doi: 10.1371/journal.pone.0301092 (PMC11078425; doi:10.1371/journal.pone.0301092)
Supplement: S2 Table — (PDF) [file pone.0301092.s002.pdf]

S2 Table Means ± SDs for the individual free amino acids and the *p* values between the healthy and patient groups

	HC	DM		HT		AGA		MDD		AD		CI	
	Mean ± SD	Mean ± SD	*p* value	Mean ± SD	*p* value	Mean ± SD	*p* value	Mean ± SD	*p* value	Mean ± SD	*p* value	Mean ± SD	*p* value
Ala	66.96 ± 83.77	182.14 ± 200.29	**<0.001**	175.99 ± 199.28	**<0.001**	137.69 ± 135.32	**<0.001**	153.13 ± 221.54	**<0.001**	510.45 ± 1093.82	**<0.001**	272.55 ± 422.76	**<0.001**
Arg	74.09 ± 92.20	129.48 ± 108.69	**<0.001**	122.71 ± 95.36	**<0.001**	139.36 ± 106.20	**<0.01**	161.20 ± 142.91	**<0.001**	196.90 ± 235.31	**<0.001**	156.55 ± 213.11	**<0.001**
Asp	244.38 ± 553.63	407.40 ± 254.33	0.087	421.65 ± 353.10	**<0.05**	378.19 ± 269.00	0.306	258.64 ± 285.99	0.909	343.13 ± 314.19	0.521	560.68 ± 390.39	**<0.05**
Cys	8.89 ± 57.29	8.06 ± 6.58	0.933	30.37 ± 147.10	**<0.05**	8.35 ± 4.54	0.968	9.57 ± 10.13	0.957	6.78 ± 4.30	0.894	25.23 ± 49.26	0.272
Cysteic acid	9.05 ± 8.26	11.84 ± 6.81	0.051	12.22 ± 10.33	**<0.05**	11.57 ± 4.35	0.197	17.48 ± 12.82	**<0.001**	13.65 ± 12.02	**<0.05**	20.10 ± 25.76	**<0.001**
Glu	388.43 ± 447.20	687.27 ± 519.00	**<0.001**	685.69 ± 606.70	**<0.001**	671.08 ± 547.72	**<0.01**	443.78 ± 502.18	0.584	431.63 ± 334.33	0.729	926.46 ± 657.42	**<0.001**
Gly	80.18 ± 86.39	225.17 ± 240.79	**<0.001**	200.18 ± 220.85	**<0.001**	134.64 ± 139.03	**<0.01**	170.62 ± 216.75	**<0.001**	689.63 ± 1398.31	**<0.001**	373.48 ± 648.49	**<0.001**
His	52.09 ± 54.58	126.55 ± 105.69	**<0.001**	113.80 ± 95.30	**<0.001**	104.04 ± 76.19	**<0.001**	125.56 ± 122.26	**<0.001**	208.56 ± 264.96	**<0.001**	155.49 ± 204.22	**<0.001**
Ile	73.53 ± 93.50	166.42 ± 141.42	**<0.001**	165.94 ± 159.26	**<0.001**	152.86 ± 128.85	**<0.001**	141.36 ± 174.66	**<0.01**	214.01 ± 360.16	**<0.001**	220.47 ± 207.04	**<0.001**
Leu	121.20 ± 167.58	283.25 ± 255.85	**<0.001**	283.22 ± 280.61	**<0.001**	273.11 ± 233.37	**<0.001**	241.46 ± 314.80	**<0.01**	332.99 ± 544.91	**<0.001**	387.60 ± 365.84	**<0.001**
Lys	51.15 ± 50.63	121.03 ± 93.27	**<0.001**	111.26 ± 90.43	**<0.001**	115.06 ± 69.74	**<0.001**	123.29 ± 93.03	**<0.001**	156.74 ± 157.21	**<0.001**	294.24 ± 466.29	**<0.001**
Met	2.24 ± 4.42	10.32 ± 17.96	**<0.001**	9.04 ± 9.97	**<0.001**	6.56 ± 8.49	**<0.001**	6.17 ± 9.09	**<0.001**	15.30 ± 27.94	**<0.001**	21.93 ± 38.33	**<0.001**
Phe	115.45 ± 135.06	242.52 ± 186.26	**<0.001**	234.47 ± 204.07	**<0.001**	238.09 ± 173.33	**<0.001**	202.28 ± 246.91	**<0.01**	265.78 ± 373.47	**<0.001**	291.57 ± 247.21	**<0.001**
Pro	29.41 ± 34.31	78.36 ± 71.65	**<0.001**	79.76 ± 90.94	**<0.001**	67.55 ± 53.04	**<0.001**	57.49 ± 70.31	**<0.001**	165.71 ± 347.55	**<0.001**	108.52 ± 113.18	**<0.001**
Ser	183.26 ± 202.63	572.83 ± 569.09	**<0.001**	556.91 ± 552.96	**<0.001**	394.12 ± 335.20	**<0.001**	474.02 ± 633.84	**<0.001**	1418.92 ± 2566.89	**<0.001**	838.88 ± 1171.01	**<0.001**
Thr	57.12 ± 65.08	149.40 ± 139.58	**<0.001**	144.46 ± 143.72	**<0.001**	115.29 ± 96.85	**<0.001**	124.60 ± 161.73	**<0.001**	291.35 ± 497.98	**<0.001**	219.25 ± 289.64	**<0.001**
Tyr	123.68 ± 126.75	242.73 ± 168.66	**<0.001**	227.18 ± 177.94	**<0.001**	231.83 ± 144.26	**<0.001**	199.94 ± 217.07	**<0.01**	241.39 ± 273.92	**<0.01**	283.67 ± 221.37	**<0.001**
Val	61.14 ± 80.46	160.64 ± 146.89	**<0.001**	158.94 ± 172.77	**<0.001**	137.80 ± 120.97	**<0.001**	134.80 ± 169.99	**<0.001**	251.48 ± 501.59	**<0.001**	221.36 ± 238.66	**<0.001**
