# Supplementary material for: Comparative studies of hair shaft components between healthy and diseased donors
Source: PLoS One. 2024 May 8;19(5):e0301092. doi: 10.1371/journal.pone.0301092 (PMC11078425; doi:10.1371/journal.pone.0301092)
Supplement: S3 Table — (PDF) [file pone.0301092.s003.pdf]

S3 Table A comparison of the effect sizes of minerals between the healthy and patient groups

	DM	HT	AGA	MDD	AD	CI
Li	0.146	0.184	0.043	0.321	0.156	0.254
Be	0.365	0.126	0.300	0.785	N.D.	N.D.
B	0.240	0.786	0.265	0.235	0.290	0.757
Na	0.350	0.223	0.158	0.109	0.722	0.521
Mg	0.586	0.467	0.557	0.080	0.320	0.123
Al	0.659	0.500	0.555	0.630	0.526	0.536
P	0.466	0.237	0.121	0.355	**1.095**	0.428
K	0.329	0.344	0.350	0.222	0.515	0.478
Ca	0.577	0.277	0.675	0.136	0.187	0.142
V	N.D.	N.D.	N.D.	N.D.	N.D.	N.D.
Cr	**0.943**	0.576	**1.215**	0.090	0.266	0.218
Mn	**0.844**	0.307	0.638	0.233	0.032	0.018
Fe	0.364	0.234	0.405	0.099	0.002	0.051
Co	0.452	0.365	**0.839**	0.097	0.015	0.028
Ni	0.021	0.024	0.014	0.094	0.115	0.081
Cu	0.253	0.114	0.267	0.192	0.249	0.257
Zn	0.205	0.008	0.043	0.061	0.414	0.332
Ge	N.D.	N.D.	N.D.	N.D.	N.D.	N.D.
As	0.611	0.426	0.218	0.194	0.531	0.494
Se	0.319	0.128	0.315	0.247	0.314	0.300
Br	0.072	0.137	0.292	0.037	0.198	0.138
Sr	0.544	0.448	0.613	0.084	0.346	0.241
Zr	0.330	0.304	0.239	0.361	0.343	0.238
Mo	0.248	0.017	0.043	0.087	0.048	0.021
Cd	0.137	0.125	0.153	0.072	0.029	0.241
I	0.631	0.496	0.585	0.382	0.549	0.618
Ba	0.025	0.028	0.031	0.079	0.011	0.047
Hg	**0.839**	0.219	0.309	0.750	0.293	0.333
Pb	0.001	0.062	0.041	0.576	0.335	0.206
