# Supplementary material for: Comparative studies of hair shaft components between healthy and diseased donors
Source: PLoS One. 2024 May 8;19(5):e0301092. doi: 10.1371/journal.pone.0301092 (PMC11078425; doi:10.1371/journal.pone.0301092)
Supplement: S4 Table — (PDF) [file pone.0301092.s004.pdf]

S4 Table A comparison of the effect sizes of free amino acids between the healthy and patient groups

	DM	HT	AGA	MDD	AD	CI
Ala	0.750	0.713	0.628	0.515	0.572	0.675
Arg	0.550	0.518	0.656	0.724	0.687	0.502
Asp	0.378	0.382	0.307	0.032	0.219	0.660
Cys	0.020	0.192	0.013	0.017	0.052	0.306
Cysteic acid	0.369	0.339	0.382	0.782	0.446	0.578
Glu	0.617	0.558	0.565	0.116	0.109	**0.957**
Gly	**0.802**	0.716	0.471	0.548	0.615	0.634
His	**0.885**	0.795	0.784	0.776	**0.818**	0.692
Ile	0.775	0.708	0.705	0.484	0.534	**0.915**
Leu	0.749	0.701	0.748	0.477	0.525	**0.936**
Lys	**0.931**	**0.820**	**1.049**	**0.963**	**0.904**	0.733
Met	0.618	**0.882**	0.638	0.550	0.653	0.721
Phe	0.781	0.688	0.789	0.436	0.535	**0.884**
Pro	**0.871**	0.733	**0.854**	0.508	0.552	**0.946**
Ser	**0.912**	**0.897**	0.761	0.618	0.679	0.780
Thr	**0.847**	0.783	0.705	0.547	0.660	0.772
Tyr	0.798	0.670	0.796	0.429	0.552	**0.887**
Val	**0.840**	0.726	0.746	0.554	0.530	**0.900**
